# Epilepsy associated tuberous sclerosis; a case report from Bangladesh

**DOI:** 10.1016/j.amsu.2022.103738

**Published:** 2022-05-11

**Authors:** Abhigan Babu Shrestha, Sajina Shrestha, Senjuti Seemanta, Pashupati Pokharel, Waqar Alam

**Affiliations:** a Department of Internal Medicine, M Abdur Rahim Medical College, Dinajpur, Bangladesh; b Department of Internal Medicine, KIST Medical College, Imadol, Patan, Nepal; c Department of Internal Medicine, Maharajgunj Medical Campus, Kathmandu, Nepal

**Keywords:** Angiofibroma, Epilepsy, Case report, MRI, Subependymal nodules, Tuberous sclerosis complex

## Abstract

Tuberous sclerosis or Bourneville's disease is a rare autosomal dominant disease affecting many organs like the brain, heart, lungs, eyes, kidneys and skin. It is characterized by neurological manifestation like epilepsy, cutaneous changes and the formation of benign lesions in multiple organs. The symptoms are apparent only in late childhood, which limits the early diagnosis in infancy. Here, we report a case of a 15 year old female child with tuberous sclerosis.

## Highlights

• Tuberous sclerosis or Bourneville's disease is a rare autosomal dominant disease due to mutation of TSC 1/2 gene.
• The goal of epilepsy treatment in TSC is to control and prevent seizures as soon as possible after diagnosing TSC.
• Along with anti-epileptic drug, other like Rapamycin are used and some newer hsp 90 inhibitors are under research.

## 1 Introduction and importance

Tuberous sclerosis complex (TSC) also called Bourneville's disease, first described by Magloire Bourneville in 1880, is a rare autosomal dominant disease associated with benign lesions that may affect many organs like the brain, heart, lungs, eyes, kidneys and skin [1,2]. This involvement thus present with different signs and symptoms. Mutation in one of the two tumor suppressor genes, TSC1 encoding hamartin, and TSC2 encoding tuberin is accountable for approximately 85% of cases of this multisystemic tumor syndrome called TSC [3,4]. The products of these mutated genes cause the deactivation of the inhibition of the mammalian target of rapamycin (mTOR) signaling cascade [5]. Hence, resulting in uncontrolled cellular growth, proliferation, and protein synthesis [6].

According to two epidemiologic studies, the prevalence of TSC was found to be 1 in 100,000 and 1 in 29,900 among patients younger than 65 years of age respectively. There are no propensity for sex and ethnic group [7,8]. Here, we present a case report of a 15-year-old female patient with distinctive clinical and radiological features of TSC. Her condition was not diagnosed earlier and was done now based on the major and minor criteria as described below. It is necessary to diagnose such cases early as possible for better outcome. So, clinicians must be mindful about such condition when a patient especially child presenting with seizure and dermatological manifestation. This case report has been reported in line with the SCARE Criteria [9].

## 2 Case presentation

A 15 years old female patient reported to the medicine department of M Abdur Rahim Medical College Hospital, Dinajpur, Bangladesh with generalized tonic-clonic seizures, each lasting for about 1 min, with a 5–10 minutes of observed relaxation phase. She had a history of seizures since 1 month of birth which was controlled with diazepam and used to accomplish her daily work but in an unusual manner. She couldn't attend school because of a lack of relevance and connectivity in her talking which indicated her to have an intellectual disability. Although there was no delay in her milestone, she was only able to speak in small phrases. Her pre and postnatal period were uneventful in accordance to her mother. She's the youngest among 3 siblings and there is no such similar conditions seen in them. None of her family members suffered from any dermatological and neurological problem.

At first, she had seizures monthly which then progressed to weekly and now with daily seizures which were difficult to control with diazepam due to which she had to be admitted.

**Fig. 1:**
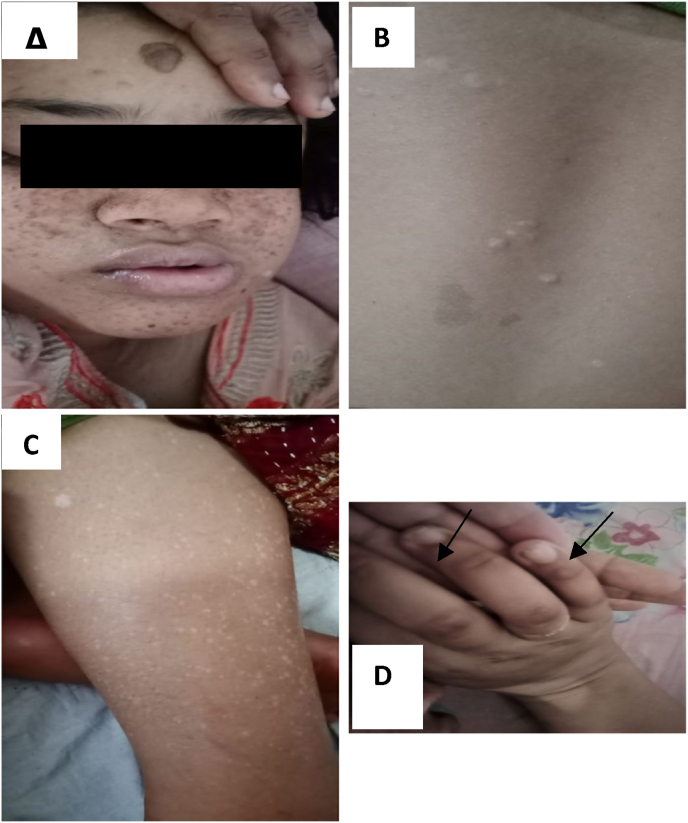
Features of Tuberous Sclerosis in the patient. Multiple angiofibroma (A). Shagreen patch (ash leaf marks) over the lumbar region (B). Multiple hypomelanotic macules over lower legs (C). Ungual fibromas over the fingers (D).

**Fig. 2:**
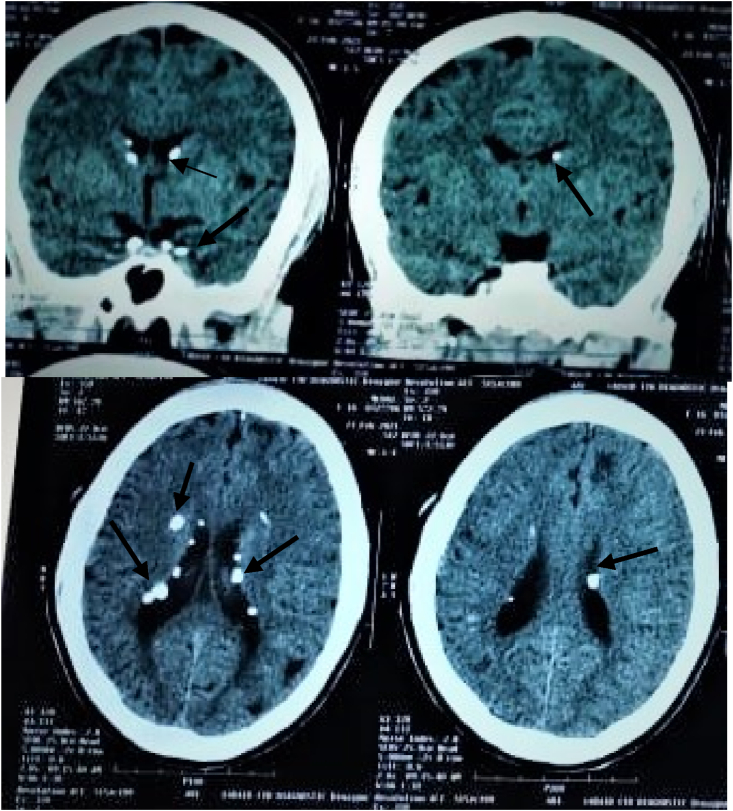
CT scan of head showing sub ependymal nodules.

On clinical examination revealed multiple angiofibromas (Fig. 1, A), appearing as well defined round to oval dark brown firm papules in a butterfly pattern over her face. A shagreen patch (ash leaf marks) was present over her back around the lumbar region (Fig. 1, B). Multiple hypomelanotic macules were seen over her lower limb (Fig. 1, C). Ungual fibromas were evident over her fingers (Fig. 1, D). On Nervous system examination, revealed increased tone of both upper and lower limbs with brisk deep tendon reflexes and Babinski's sign bilaterally positive but superficial reflexes were absent. Other systemic examinations were normal. Complete blood count, hemoglobin, kidney and liver function tests were normal. Cranial MRI was performed which showed multiple subependymal nodules (Fig. 2). Five of the major criteria supported the diagnosis. Due to financial restrictions and lack of facilities genetic analysis couldn't be performed.

For the management of her condition, we started with levetiracetam (500 mg b. i.d) and for the symptomatic treatment haloperidol (1mg b. i.d) and procyclidine (2.5mg t. i.d). But sometimes the seizures used to reoccur again despite the treatment, so chlorpromazine (25mg I. M) was used. Her condition stabilizes only during the medication after which seizure again reappears. We also took consultation from the neurosurgeon, but there was no surgical emergency required. Due to lack of facilities and inability to manage her condition we had to refer her to National Institute of Neurosciences and Hospital, Dhaka. Unfortunately, the follow up of the patient couldn't be recorded.

## 3 Clinical discussion

The clinical presentations and pathological manifestations of TSC vary significantly due to the involvement of multiple organs like the brain, skin, kidneys, lung, heart, and eyes [10]. Typical clinical features of TS, called the Vogt triad, comprises of epilepsy, mental retardation and facial angiofibroma, nevertheless 50% of the patient have normal intelligence and 25% do not have seizure [11]. Major criteria [10,11]:

• Angiofribroma or forehead plaque (3 or more)
• Hypomelanotic macules (3 or more atleast 5mm diameter)
• Ungual fibroma (2 or more)
• Shagreen patch
• Multiple retinal hematomas
• Multiple cortical tubers
• Subependymal nodule (2 or more)
• Subependymal giant cell astrocytoma
• Cardiac rhadbomyoma
• Lymphangioleimyomatosis
• Angiomyolipomas (2 or more)

Minor criteria:

• Dental enamel pits (more than 3)
• Intraoral fibromas (2 or more)
• Nonrenal hamartomas
• Retinal achromic patch
• Confetti skin lesion
• Multiple renal cysts
• Sclerotic bone lesions

Besides these, other neurologic manifestations, such as seizures, cognitive disability, autism spectrum disorder, behavioral issues, hamartomatous rectal polyps, high arched palate, bifid uvula, cleft palate, delayed dental eruption, and presence of diastemas are other common presentations [12,13]. Patients presenting two major criteria, or one major and two minor criteria are diagnosed with definite TSC based on genetic testing [11]. In this case 5 of the major criteria testified our diagnosis.

A retrospective study by Catherine J. Chu-Shore between 2002 and 2008 states that 246 patients had developed epilepsy out of 291 patients, denoting epilepsy as the most common neurological manifestation of TSC patients [14]. After the first seizure episode of TSC patients, about 100% of them have the probability of developing epilepsy [16]. So, it is quite important for physicians to monitor the patient cautiously. Usually, TSC related epilepsy is common from childhood, in most cases in the very first months, but it also may appear at the later phase of life [1]. Though the most common pattern of seizure is a focal seizure, it can alter eventually in the future [15].

A cohort study about epilepsy in children with TSC at a tertiary level hospital of Bangladesh states that 37% of patients had an onset of seizure in the first six months of life [15]. The generalized seizure was second-most, and focal seizure was the most common pattern of the seizure [15]. More than half of the patients in the cohort received vigabatrin either as a single agent or given in combination with other AED (anti-epileptic drugs). Only 34.2% of patients had freedom from sustained seizure, while 4.2% had a relapse [15]. In contrary, with smaller sample size, still there is loop hole to be filled for proper overall management guideline for rare disorders and diagnosis. In our case, patient wasn't diagnosed earlier and now required multiple AED to become stable. The choice of drug depends on how early we catch the diagnosis.

Due to vague manifestation, the diagnosis and treatment approaches from multiple disciplines must be considered prior to treatment planning as well as suitable and effective therapy should be provided to ensure the best outcome with lesser adverse effects and large-scale benefits for the patient [11,13].

There is no definitive treatment for TSC, for seizure control GABAergic drugs (i.e. vigabatrin) are used as the first-line drug to control focal seizures before the age of one year [1]. Whereas, Levetiracetam; an antiepileptic therapy in patients with TSC can reduce seizure frequency [13]. Sodium valproate, phenobarbitone, benzodiazepine, oxcarbazepine, carbamazepine, topiramate, and oral steroids are used for symptomatic treatments if needed [13]. More than one drug is used if necessary in patients with TSC [1]. In our case we had to use multiple AED to control epilepsy and the drugs were used based on the availability and economic condition of the patient. Surgery is usually not done but the presurgical evaluation is recommended after the failure of a combination of two AEDs or in infants with large dysplastic lesions [11].

Systemic everolimus (mTOR antagonist) is another drug that effectively reduces or prevents the growth of subependymal nodules and improves skin lesions like facial angiofibroma [16]. Everolimus also improves autistic behavior, concentration, hyperactivity, and depression related to TSC [12]. Topical sirolimus and everolimus are a preferable option for facial angiofibromas nowadays as it has less adverse effects than the systemic ones [17].

Rapamycin, a commercially available immunosuppressant, and FDA approved drug, is effective for TSC related facial angiofibromas when used topically [1,5]. Oral rapamycin therapy can be used as an alternate option for surgical procedures if the response is adequate [8]. A ketogenic diet (anticonvulsant effect of ketone bodies), vagal nerve stimulation, fructose derivatives, neurosurgical intervention are also other alternative approaches in the treatment of patients with TSC [11,16].

In a mouse model, it was observed that the loss of TSC1/2 protein activity in neurons lead to a reduction of cilia. To their finding, same results were seen on giant cells in cortical tubers brain samples from patients with TSC. Which established a relation between TSC1/2 with loss of cilia. They proposed an alternative target to mTOR inhibitors like rapamycin, HSP 90 inhibitors. The rapalogs provide some benefit for treatment of TSC associated epilepsy and tumor, and are ineffective for neuropsychiatric symptoms. On the other hand, HSP 90 inhibitors might be potentially more effective and safer than rapalogs [18]. This treatment might be the light at the end of the tunnel.

The prognosis depends on the severity or different organs involvement. In severely affected infants, 25% die before the age of 10 years and 75% before 25 years. However, if diagnosed late in life with fewer cutaneous signs, prognosis rely on the associated cerebral calcifications and tumor size and location [19].

The strength of this article is that it has included the treatment for TSC including the latest ongoing HSP90 inhibitor drug target. It has showcased importance of early diagnosis which is crucial for better outcomes and quiet important in developing countries like Bangladesh. The weakness of this paper is that the treatment efficacy can't be gauzed by a single patient. Even larger cohort studies should be conducted to decide proper management guidelines.

## 4 Conclusion

TSC is a multisystem disorder presenting with a wide spectrum of clinical presentations affecting many organs, which could be diagnosed in early life with the cutaneous presentation and radiological techniques. The goal of epilepsy treatment in TSC is to control and prevent seizures as soon as possible after diagnosing TSC, which could improve cognitive neurodevelopment and have a positive impact on the quality of life.

## Ethical approval

N/a.

## Sources of funding

none.

## Author contribution

All authors contributed equally in this article.

## Trial registry number

1. Name of the registry: N/a
2. Unique identifying number or registration ID: N/a
3 Hyperlink to your specific registration (must be publicly accessible and will be checked): N/a

## Guarantor

First author.

## Consent

Written informed consent was obtained from the parents of the patient for publication of this case report and accompanying images. A copy of the written consent is available for review by the Editor-in-Chief of this journal on request.

## Provenance and peer review

Not commissioned, externally peer-reviewed.

## Declaration of competing interest

none.
